# The Change of Life in Women, and the Ills and Ailings Incident Thereto

**Published:** 1899-10

**Authors:** 


### BOOK NOTICES.

THE CHANGE OF LIFE IN WOMEN, AND THE ILLS AND AILINGS INCIDENT THERETO. By J. Compton Burnett, M. D. Philadelphia: Bœricke & Tafel, 1898. Price, cloth, $1.00; by mail, $1.06.

Dr. Burnett is well known to the members of our school of medicine for his numerous autographs on special medical subjects. These little works, written in his charming style, are most instructive and inspiring to the homœopathist and stimulate him to an emulation of his methods and his successes.

His explanation of the menstrual function is admirable and we herewith reproduce it for the benefit of our readers.

"The girl's digestion and assimilation are so arranged that she shall for some thirty years or so of her life's course, from puberty to menopause, make blood enough for her own maintenance and activities plus what should or might be needed for gestation and lactation, the menstruation being primarily a means of maintaining her equipoise by throwing overboard at stated times a given not-called-for blood supply, prepared by the time of each ovulation as a possibly needed food reserve, which throwing overboard of said supercargo does not occur if impregnation of the ovule takes place. It is this menstrual arrangement which must be kept in view if we are to understand the change of life and its sequels and indeed if we are to understand women's diseases at all at any period."

At page 18, he speaks of flushes of heat, for which his principal remedy is Lachesis. After that he prefers Urtica-urens, and also speaks favorably of Glonoine. Leucorrhœa he regards as a "constitutional outlet for many morbid products;" and concludes that the use of injections for its suppression is "utterly bad, a downright sin against nature's ways." He considers injections a "stupendous error and fraught with untold evil consequences, and nasty and vulgar to boot."

Here is a lesson we all need to learn and it is likely to be remembered clothed in the foregoing forceful words. On pages 28 to 30, he relates a case where the suppression of the leucorrhœa by injection gave most prolonged sickness and suffering to the patient.

In the view of the author, the change of life ought to be perfectly free from pain if the patient be healthy. This view is that of older masters of Homœopathy like the late Dr. Henry N. Guernsey.

Tumors of the breasts and womb appearing about the time of the change of life are regarded by Dr. Burnett as representing the accumulation of pre-existent constitutional taint that previously found its outlet in the menstrual flux and in leucorrhœa. He then proceeds to give a case where a cured was effected.

He finds from his own experience, that the itch is not curable by homœopathic remedies. It is necessary to destroy the itch insect by germicides if we are to succeed. Then he would treat the *effects* of the psora with homœopathic remedies. The foregoing give the main points of value in the book and enable the reader to form some idea of its contents. No review can do these little books of Dr. Burnett justice. Every physician should have a full set of them and read with a view to remember all they contain.

Dr. Burnett would be doing something both sensible and serviceable if he would publish a repertorial index or concordance repertory to the whole set; each indication for a remedy having a reference to volume and page.

